# Knowledge, Awareness, and Practices toward Colorectal Cancer and Its Dietary and Lifestyle-Related Risk Factors among Jordanian University Students: A Cross-Sectional Study

**DOI:** 10.1155/2024/4503448

**Published:** 2024-02-15

**Authors:** Husam Khraiwesh, Dana N. Abdelrahim, Iman F. Mahmoud, MoezAlIslam Faris

**Affiliations:** ^1^Department of Nutrition and Food Processing, Faculty of Agricultural Technology, Al-Balqa' Applied University, Salt, Jordan; ^2^Research Institute of Medical and Health Sciences (RIMHS), University of Sharjah, Sharjah, UAE; ^3^Department of Nutrition, Faculty of Pharmacy and Medical Sciences, University of Petra, Amman, Jordan; ^4^Department of Clinical Nutrition and Dietetics, College of Health Sciences/Research Institute for Medical and Health Sciences, University of Sharjah, Sharjah, UAE

## Abstract

**Background:**

Globally, colorectal cancer (CRC) incidence is rising, and it is a leading cause of mortality, with greater death rates pronounced in developing countries, including Jordan. Understanding knowledge and awareness of etiologic factors, unhealthy lifestyles, and dietary patterns is crucial for combating ailments. Hence, this study is aimed at investigating the level of knowledge and awareness of CRC-related risk factors, practices, and possible associations of studied variables among young Jordanians. *Methodology*. A cross-sectional, observational study was conducted using an online self-reported assessment of anthropometrics, knowledge, awareness, and dietary and lifestyle practices toward CRC and its related risk factors.

**Results:**

A study of 795 Jordanian university students found that 93.8% were Jordanians, 73.0% were female, aged 18-24, and single. Most participants were from medical and science schools (69.4%). The vast majority (about 84%) were found to have good knowledge and awareness of CRC and its risk factors, but this was not reflected in their dietary practices. There are significant differences in physical activity, smoking, vegetable consumption, and serving sizes of red meat and processed meats between the sexes. Academic study specialties significantly impact knowledge and awareness.

**Conclusion:**

The study reveals that while young Jordanian university students have good knowledge and awareness about CRC and its risk factors, these levels are not reflected in their dietary behaviors and food choices for CRC prevention, highlighting the need for national programs to improve these practices, particularly in the younger population.

## 1. Introduction

Worldwide, colorectal cancer (CRC) is a leading cause of morbidity and mortality due to its rising incidence [1]. The worldwide burden of CRC is anticipated to rise by 60% in 2030 [2]. In 2022, CRC caused 935,173 deaths worldwide, accounting for almost 10% of all cancer fatalities, with greater deaths (52%) happening in less developed nations [3]. A higher prevalence of CRC has been recorded in highly developed nations, mainly Western countries. Yet, increased CRC incidence has been noted in low and middle countries, particularly the Arab countries in the Middle East [4, 5]. In Jordan, based on the latest Jordan Cancer Registry report by the Jordanian Ministry of Health, CRC is the second most frequently diagnosed cancer for both sexes and the second leading cause of death among Jordanians [6].

The epidemiological data of CRC fluctuate throughout time and in various geographic areas and may reflect varying exposure to risk factors [7]. Several risk factors for developing CRC were reported by the World Cancer Research Fund (WCRF) and the American Institute for Cancer Research (AICR) Continuous Update Project report [8]. Despite the vast knowledge regarding CRC and its considerable effectiveness and advances in its treatment options, the absolute eradication of this disease is challenging due to its complex etiology [3]. Mounted evidence has shown a significant correlation between multiple risk factors and the initiation and manifestation of CRC [9]. These risk factors can be of a nondietary factor nature as genetic predisposition [10], coexisting chronic diseases (e.g., diabetes) [3], aging [10], physical inactivity, smoking [11], and dietary nature represented by incorrect dietary habits such as low fruits and vegetables consumption [12]; inadequate intake of whole grains [13] and dietary fibers [14], or high red meat diets [15], processed meats, fats; and excessive alcohol consumption [16].

Previous studies conducted in the Middle East and Gulf countries have reported unsatisfactory knowledge and awareness among the public regarding CRC risk factors. Indicating a need for future correction plans based on dietary behavior. For instance, Middle-aged UAE people displayed insufficient knowledge about CRC risk factors, warning signs, and screening methods [17]. Similar to Kuwait's findings [18], the public in Syria and Lebanon also lacked awareness of CRC-developing risk factors [19, 20].

In Jordan, few studies have investigated the risk factors and the general knowledge about CRC [21–24]. Some studies in Jordan have figured out the knowledge and awareness of CRC, specifically its role in screening and early detection [22, 25]; other studies searched the knowledge and awareness of modifiable CRC-related lifestyle risk factors in mid-age subjects but without its associated practices [21, 23, 24], which is considered to be the knowledge assessment's most crucial objective, so our work novelty is filling the gap in the area as there has not been a similar study in Jordan.

Taha et al. [21] and Omran et al. [26] found low knowledge of colorectal cancer (CRC) in public, with an underestimation of perceived CRC risk. Around 6% of participants suggested modifying dietary and lifestyle behaviors to reduce CRC risk, but correct responses ranged from 5.6% to 28.9% when considering potential risk factors. This indicated poor knowledge regarding CRC risk factors among Jordanians [26].

In addition, the risk of developing CRC is well-documented to be higher in older people over 50 years. However, recent studies estimated that around 1 out of 4 rectal cancers and 1 out of 10 colon cancers are detected in adults under 50 years [27–29]. Jordan is considered one of the youngest populations globally, with more than half of the population categorized under the age of 30 years [30, 31]. This young population is more prone to a high risk of CRC due to the adaptation of Westernized diets and unhealthy lifestyles [32], which are positively correlated with higher CRC incidence [33, 34]. Hence, an educational intervention aimed toward the prevention of diseases coming with older age earlier in young adulthood is the most beneficial. Though CRC has gained increasing attention in Jordan, up until today, there is a massive need for more studies assessing the level of knowledge, awareness, and relevant practices toward the prevention of CRC.

Improving knowledge and awareness of the risk factors plays a vital role in lowering the burden of this disease [22]. Moreover, nationally designed programs to increase knowledge, awareness, and correct practices are to date, absent, or insufficient [21]. Hence, the present study is based on the hypothesis that university students in Jordan are likely to exhibit a prevalence of poor levels of knowledge and awareness and unhealthy dietary and lifestyle practices related to CRC etiology. Together with the undeniable correlation between poor dietary and lifestyle choices and CRC occurrence, especially among young adults such as university students, this study was designed to investigate the levels of knowledge, awareness, and practices regarding the role of dietary and lifestyle risk factors in CRC among Jordanian university students and to find out the relation between KAP components and the sociodemographic variables.

## 2. Materials and Methods

### 2.1. Study Protocol

The current work followed the guidelines of Strengthening the Reporting of Observational Studies in Epidemiology (STROBE) in reporting the study design and results [35]. This study is a cross-sectional study that was conducted between October 2022 and November 2022. A nonprobability convenience sample was collected for undergraduate and graduate students in various Jordanian universities. Online invitation letters were sent to all university students as part of the recruitment process by official links related to the targeted universities, where most university students were available. A link to the online survey was included in the invitation letter. Before participants could continue with the computerized questionnaire's second page, they were given information about the study objectives and asked for their approval to participate in this study. The inclusion of the participants was done primarily through a nonprobability convenience sampling technique. The study included all accessible undergraduate and postgraduate students attending Jordan universities during the academic year of 2022/2023.

### 2.2. Data Collection Tools

An online, electronic questionnaire with two domains was used; a pretested one was used to assess the knowledge and awareness of the role of dietary and lifestyle factors in CRC [36], and another valid questionnaire [37] was used to assess dietary and lifestyle behaviors; both domains of the questionnaire were pretested before administration pilot testing with 35 students from the same targeted group, and it was edited according to the feedback obtained. The online link was publicly shared on a specialized fakebook page for Jordanian university students with 595,000 members. This number is close to the country's total number of university students.

Participants were given a brief explanation of the study and its main purposes. The questionnaire started with questions related to participants' sociodemographics, including age, sex, marital status, nationality, educational level, living place, and monthly allowance. The main body of the questionnaire included three domains: (1) anthropometric measurements, self-reporting of body weight and height and lifestyle characteristics, physical activity patterns, and smoking status; (2) participants' dietary behaviors summarized in around 20 food groups and are considered associated CRC risk factors; and (3) knowledge about CRC and its related modifiable (e.g., dietary and lifestyle) and nonmodifiable (e.g., age and sex) risk factors.

### 2.3. Data Identification

From the data of the self-reporting of body weight and height, the body mass index (BMI; kg/m^2^) was calculated and categorized based on WHO classification: underweight (less than 18.5), normal weight (18.5–24.9), overweight (25.0–29.9), and obese (30 and above). As for the lifestyle risk factors, data was obtained from questions about the physical activity levels and the smoking status. The physical activity questions included how often participants engage in physical activities (e.g., walking, swimming, gymnastics, football, and aerobics). The options provided were sedentary physical activity levels (less than 30-60 min, once a week or do not practice exercise at all), moderate physical activity levels (30-60 min, 1-3 times per week, or 30-60 min, 4-6 times per week), and high physical activity levels (30-60 min daily, or more than 30-60 min daily). The amount of time was taken from the WHO's global recommendations for adults' physical activity for health [38].

The dietary information included the frequency and quantity of consumed foods related to CRC prevention and initiation, such as fruits, vegetables, dietary fibers, onion, dietary fats, red meats, processed meats, dairy products, and French fries. Moreover, the usual portion sizes of these food items were assessed using the options “never, one serving,” “2-3 servings,” and “4 or more servings,” while the frequency questions have five choices ranging from never to 1-2 times daily. The food group portion size examples were elaborated after each question using two-dimensional photos and word descriptions (see Table 1 for the questionnaire details).

Based on the participants' replies to 18 specific questions, their choices and agreement or disagreement with the significance of particular risk factors linked to CRC were used to assess their knowledge and awareness of CRC and its risk factors. In the first four questions, every correct answer scored 1, while all wrong answers received a 0. Fourteen risk factors were given using a Likert scale of strongly disagree, disagree, neutral, agree, and strongly agree in determining the knowledge about dietary and lifestyle risk factors associated with CRC. Responses to agree or strongly agree were scored 1, while the rest were given 0. An overall score of knowledge and awareness was calculated by summing all scores from all 18 questions in the section, after which, for improved interpretability, a percentage of the overall score was calculated [39]. The overall score was defined as poor knowledge, considered for a total point of less than 9, obtaining less than 50% out of 18 questions as correct responses, while scores equal and above 9 indicated good knowledge and awareness levels about CRC and its risk factors.

### 2.4. Statistical Analysis

The categorical variables were described using frequencies and percentages of observed values. Sociodemographic variables were transformed into two categories as follows: sex (male vs. female), age (18-24 years vs. 25 years and above), marital status (single vs. married, divorced, and widowed), nationality (Jordanian vs. non-Jordanian), specialty (medical and scientific vs. nonmedical), educational level (postgraduate vs. undergraduate), living place (in campus vs. out campus), and monthly allowance (less than or equal to 50 JD vs. more than 50 JD). All risk factor behaviors of the participants were categorized into binary choices, risky or protective, to assess their correlation with their knowledge levels. KAP components were considered as the outcomes (dependent variables), while the sociodemographic variables and dietary and lifestyle behaviors were considered as exposures (independent variables). The cross-tabulation and Pearson's chi-squared tests were performed to find the odds ratio (OR) and the 95% confidence interval (CI) of the associations between all categorical variables and the total knowledge score. Participants' responses were encoded and analyzed using IBM SPSS statistics, version 26.0 (USA). The significance level for all collected data was set at a *P* < 0.05.

### 2.5. Study Ethics

Ethical approval was obtained from the research ethics committee (REC) of the UOS (REC-20-05-26-03-S). Participation was voluntary; only students from both sexes aged 18 years and above, from both medical and nonmedical colleges who were willing to participate and agree on the consent form, were included in the study. No monetary or nonmonetary incentives were given to participants.

## 3. Results

The sociodemographic, educational, anthropometric, and lifestyle characteristics of the study participants are presented in Table 2. A total of 795 students participated in the study, with female students being the dominant number (73.0%) compared to male students (27.0%). Most participants were within the age group of 18-24 years (85.3%). Almost all participating students were Jordanian (93.8%). Most participants were single (90.4%) and lived outside campus with their families (92.2%). Around 30% of the study participants were poor and had an allowance of less than 50 JD (37$) monthly. Most participants were from medical and science schools (69.4%). The study's participants were mainly in their second (24.5%) and fourth (37.6%) academic years. The average weight was 65.04 ± 14.82 kg, average height was 165.13 ± 11.03 cm, and the average BMI was 23.76 ± 4.36 kg/m^2^. Based on the WHO BMI categorization, around 63.9% of the participants were in the normal body weight category. About 18.7% of participants were physically inactive, and 43.5% were smokers.

Table 3 describes the frequency and quantity of each food item based on participants' self-estimation.

### 3.1. Knowledge of CRC and Awareness regarding Risk Factors

Table 4 lists the participants' ratings of their understanding of CRC and its dietary and lifestyle risk factors. The question about sex is “Which group of people do you believe is more likely to develop CRC?” Less than half (33.3%) of the participants answered it correctly [40]. Almost all (96.6%) of the study's participants and the majority (63.0%) responded correctly to the question “Do you think that diet plays a role in causing CRC?” and “Do you think diet plays a role in preventing CRC?”, respectively. Meanwhile, the question “Do you believe that CRC is hereditary?” was correctly answered by half of the participants (51.1%). The following percentages represented the correct answers provided by those who strongly agreed or agreed regarding CRC risk factors: 61.1% agreed that having a family history of CRC increases its risk. In comparison, 70.1% believed that being physically inactive increases CRC risk, and 47.9% agreed that diabetes increases CRC risk. As for certain nutritional factors and their link to the incidence of CRC, 81.8% of participants believed that CRC risk increases with frequent consumption of alcohol, frequent high-fat intake (79.7%), frequent intakes of high-calorie food (73.5%), low fiber intakes (72.6%), obesity as a risk factor (72.1%), smoking (76.5%), frequent consumption of red meat (51.3%), frequent consumption of processed meats (77.9%), low vegetable and fruit intakes (76.7%), and high-stress levels (77.9%). A mean score of 12.46 ± 3.93 out of 18 was found for the total knowledge score, accounting for the vast majority of study participants (83.9%) who had good knowledge and awareness, while 16.1% had poor knowledge and awareness of CRC and its risk factors.

### 3.2. Participants' Dietary Practices and Lifestyle Behaviors

A significant difference was observed for the physical activity levels as the proportion of male participants reported more than three days/week than that reported by female participants (*P* = 0.0001). Male smokers were significantly higher than females (*P* = 0.0001). Moreover, the consumption of red meats (*P* = 0.0001), and processed meats (*P* = 0.021) were reported at significantly higher rates among females compared to males. On the other hand, the female participants reported consuming more than three servings of vegetables/per day, significantly higher than the males (*P* = 0.0001). The actual amount of intake was obtained by multiplying frequency with quantity and then categorizing them into (risky/protective) categories based on the definitions in Table 5 for each food item.

### 3.3. Associations of Participants' Sociodemographic Characteristics, Knowledge and Awareness Level, Score, and Practices regarding CRC and Its Risk Factors

The risk assessment of CRC knowledge and awareness and the sociodemographic data, dietary, and lifestyle behaviors are reported in Table 1. 53.0% (OR = 0.526, 95% CI 0.333-0.829) of students from science or medical schools were likely to have poor knowledge (*P* = 0.005). A 58.0% (OR = 1.581, 95% CI 0.995-2.514) more significant probability of insufficient appropriate knowledge was linked to inadequate consumption of dairy products.

## 4. Discussion

Community-level lifestyle modification programs can be successfully implemented [41] by significantly influencing knowledge and awareness of habits and patterns among various groups, especially the young population, which are crucial for their future proper practices [42] Hence, this study is aimed at evaluating Jordanian university students' understanding and practices regarding the role of dietary and lifestyle risk factors in CRC.

Intriguingly, our findings showed that most of the study's participants, Jordanian students, had good knowledge and awareness regarding CRC and its dietary and lifestyle risk factors. This agreed with a previous study conducted in the UAE, where almost 93% of participants showed good knowledge scores regarding CRC risk factors [36]. However, in our study, the knowledge and awareness level were not reflected in the dietary behavior of the students.

Youth knowledge and awareness alone are not the objective but a means for combating a disease; evaluating the means may be insufficient due to the possible barriers to achieving changing behavior toward CRC-related risk factors as a main goal. Hence, what distinguishes our work is measuring the behavior of the subject for each risk factor as a final goal to highlight the possible causes of practices inconsistent with acquired awareness or any other challenge.

Moreover, the results of this study showed higher knowledge and awareness levels among participants than those reported in previous studies done in the Middle East, including Jordan [19, 21, 24, 43, 44]. For instance, our results contradict the results of both Taha et al. and Mhaidat et al., who found that CRC knowledge among Jordanians was either fair or poor [21, 23], and contradicted other studies conducted in the Mediterranean Basin [19, 43] but were in line with our results in which there is are high knowledge and awareness levels among participants regarding CRC and its risk factors [24]. Furthermore, a similar pattern of results was obtained in the study by Almutlaq et al., who reported fewer shared modifiable risk factors in their research and reported less knowledge regarding the risk factor of mental illness (60.7%) compared to the stress factor reported in this study (77.9%) [44].

The contradicting results of this study with previous studies may be explained by the discrepancies in targeted participants' age and educational level, scoring model, as well as the use of assessment tools for the thoroughness of lifestyle-related risk variables and the conception of CRC awareness, where some researchers included certain additional diseases as risk factors [19]. Another possible explanation for inconsistencies in reports is the time at which these studies were conducted, particularly those conducted in Jordan before and after the COVID-19 epidemic. During the COVID-19 epidemic, many participants had the opportunity to spend more time on media, which was the primary source of their health knowledge [24, 45, 46].

Nonetheless, the findings of this study were consistent with those reported by Hashim et al. [36]. The average knowledge and awareness of the participants about modifiable factors (73.6%) were more than nonmodifiable risk factors (40.8%). This was nearly equivalent to the results found in Hashim et al.'s research, where they reported 72.8% knowledge and awareness about CRC modifiable factors and 38.7% regarding its nonmodifiable risk factors. The knowledge and awareness score discrepancies between modifiable and nonmodifiable risk factors also agreed with previous studies conducted in Jordan [23] and those reported in Arab countries like Saudi Arabia [44] and Lebanon [19]. For instance, only 51.9% of the participants in our study were aware that becoming older may be a risk factor for CRC. A similar percentage was found in both studies by Tfaily et al. and Almutlaq et al., where they reported that 48.6% and 48.9% of the participants agreed that age is one of the CRC risk factors [19, 44]. Even though younger populations seek more knowledge regarding the benefits of healthy eating than general health, the acquired knowledge sometimes needs to be corrected or added [47]. Ashraah et al. reported a low percentage of university students about the knowledge and awareness of illnesses that were incorrectly assumed to be sex-related (33.3%) [48]. This was similar to our study, where less than half of the participants knew that CRC risk was equally found in both sexes. Participants in our study and Hashim et al. agreed that frequent alcohol and fat consumption were the most often acknowledged possible risk factors for CRC. Alcohol was considered the most risk factor for CRC known among participants in various studies conducted in Oman, Jordan, and Kuwait [18, 23, 49].

The population's awareness of sex as a risk factor for CRC was the least known factor among the factors (33.3%), which was in line with that found by Althobaiti and Jradi (48.8%) [50] but higher than what was reported by Taha et al. (20.3%) [21]. Less than two-thirds of participants were aware of the role of family history or genetics in CRC, which was more than found in a study conducted in Saudi Arabia (20.9%) [51] and less than that reported by Althobaiti and Jradi (77.59%) [50] and Saeed et al. (73.5%) [18]. Participants were aware that obesity was a risk factor for CRC (*Do you think that obesity is a risk factor for CRC?*). 72% responded with the correct answer, showing a good knowledge regarding obesity and CRC. This result ties in well with the findings of Hashim et al. [36] and Ahmad et al. [24], but lower than those reported by Saeed et al. [18], Mhaidat et al. [23], and Tfaily et al. [19]. The prevalence of diabetes in Jordan is increasing steadily, where the overall aged-standardized prevalence increased from 14.2% in 1994 to 23.7% in 2017 [52]. It is well-documented that diabetes is one propounding risk factor for CRC. Hence, it is crucial to examine the knowledge and awareness regarding this factor [3]. Less than half of this study's participants recognized diabetes as a risk factor for CRC. This was in alignment with other studies conducted in AUE, Lebanon, Jordan, and Kuwait [18, 19, 23, 36]. Another least recognized CRC dietary risk factor detected in this study was red and processed red meat consumption, where merely half responded correctly. This is similar to the results obtained by Ahmad et al., Hashim et al., [24, 36], and Abdellatif et al. [53], who found that Jordanian students “were less knowledgeable regarding red meat consumption and health, though most participants were frequent meat eaters [53].” Awareness toward the role of physical activity in CRC prevention in this study was almost similar to that reported in a UAE study (73.2%) [36] and higher than reported in a study done in Saudi Arabia (62%) [44]. Among the demographic characteristics, no significant differences were found in knowledge and awareness levels based on age, which is consistent with what Mhaidat et al. [23] and Taha et al. [21] found. Medical and science students were significantly more knowledgeable than humanities college students regarding CRC and its risk factors. This may be ascribed to the role of academic performance, where those high secondary school average students have more chance to attend medical and science schools in the university than other majors in Jordan. This finding was revealed by Bany Sayd [54], who showed that students of science majors scored higher in nutrition awareness compared to their colleagues in the humanities majors. In this study, smoking was more prevalent among male participants than females, similar to the findings of Elshami et al. in Palestine [55]. Smoking is more accepted culturally in Jordan since this behavior is affected by practice and surroundings [56]. The same trend regarding physical activity, where male students were significantly more active than females, which is consistent with reports by Barghouti et al. [57] and Sbaih et al. [58], where male students have no social restrictions and have the ability to carry out outdoor activities; in addition to that, they are more interested in getting fit than females. Although the vast majority of the participants consumed the recommended number of servings of red meat, fat, and processed meat, female participants significantly consumed more red meat, which is contrary to what was found by Orman et al. [26], who reported that male participants consumed more red processed meat and vegetables, vegetables, and processed meat preparation is an easy way to snack at home.

Regarding the dietary factors and their association with CRC risk, these factors may act as risky or preventative factors [59, 60]. Students' practices toward nutritional risk factors are not similar to the studied food items, regarding meat, processed meat, fats, and French fries, which are considered high risk for CRC development, according to Schwingshackl et al. [61]. In Jordan, most consumed processed food is of poultry origin, as chicken is considered the most popular meat source and much cheaper than red meat [62]. Most of the students' daily consumption servings of fruits, vegetables, and fiber foods were characterized by low intakes that were considered risky [12], which are consistent with local [63] and global [64] studies. These findings are inconsistent with other studies that showed a positive correlation between high levels of general nutritional knowledge and fruit and vegetable intake [65]. Even though the primary knowledge and awareness scores were good, it is worth noting that the scores of these modifiable dietary and lifestyle risk factors have not demonstrated statistical differences in the study's participants toward each risk factor separately. Nevertheless, dairy products were the only food group by which participants were unaware that their insufficient intake was linked to a higher risk of CRC [66]. However, 58% of the students unhealthily [67] consumed less than or equal to one serving of dairy products daily. Similar findings were reported in a local and global study [63, 68]. The finding of this study showed a gap between participants' acquired knowledge and awareness toward CRC and its related dietary and lifestyle risk factors on the one hand and the practices toward certain protective food items (high-fiber foods, fruits, and vegetables) on the other. Since many studies positively linked high nutritional knowledge with dietary practices [65], assessing knowledge and awareness of modifiable dietary risk factors for cancer is the first step in fighting the disease, as the common intervention is education [69]. At the same time, knowledge of the public is not the only determinant of dietary and lifestyle practices; what is culturally available out of home plays a crucial role, too [70]. This may explain previous studies' weaknesses or lack of correlation between knowledge and practices [71]. Therefore, nutritional practice assessment is a tool for evaluating the impact of formal nutritional education programs. As Jordan still lacks a formal educational program for fighting CRC, this may explain the poor reflection of unofficial acquired knowledge in adhering to a protective diet toward the CRC [72].

## 5. Recommendations

It is vital to promote essential education that improves nutritional-related knowledge and awareness through proper channels, mainly through effective social media, which is the main harbor of nutritional information for the younger generation. Also, embedding national healthy nutrition programs for the public is considered of great importance. Furthermore, it is recommended to introduce compulsory university courses to optimize the nutrition knowledge and awareness levels and their practices for various university students to accomplish an integrated image of the students' nutrition knowledge, awareness, and healthy practices in Jordan.

## 6. Study Limitation

This study used a self-report questionnaire; this type of data collection method is subject to reporting bias from participants. Some data recalled and provided by participants may not be so accurate. Although the study included a large sample size, the convenient sampling methods may have limited the study's generalizability. Further, the lack of equality between males and females, medical and nonmedical students, may entail a source of bias. Lastly, the current findings should be tackled cautiously, considering the lack of causality owing to the observational nature of the current work.

## 7. Conclusion

In conclusion, this study shows good knowledge and awareness regarding CRC, its risk factors, and its high prevalence among young Jordanian university students. However, these levels of knowledge and awareness were not reflected in dietary behaviors and food choices in the context of CRC prevention and causation. Students face a significant barrier in adopting lifestyle practices. This can be attributed to an unofficially acquired knowledge and awareness of CRC or the influence of the environment where they spend most of their time.

The findings of this study further indicate the need for multiple national programs that promote and enhance the translation of knowledge and awareness regarding CRC and its risk factors into meaningful, healthy dietary and lifestyle practices, particularly in the younger population. Such programs would significantly reduce CRC prevalence and, hence, the mortality rates in the Jordanian community.

## Figures and Tables

**Table 1:** Sociodemographic, educational, anthropometric, and lifestyle characteristics of the study participants (*N* = 795).

Variable		Frequency	Percent (%)
Sex	Female	580	73.0
	Male	215	27.0

Age group (year)	18-24	678	85.3
	25-30	67	8.4
	>30	50	6.3

Marital status	Single	719	90.4
	Married	72	9.1
	Divorced	2	0.3
	Widowed	2	0.3

Living place	In-campus (dorms)	29	3.6
	Out-campus with family	733	92.2
	Out campus with friends	33	4.2

Monthly allowance (JOD/month)	Less than 50 JD	239	30.1
	60-100 JD	296	37.2
	More than 100 JD	260	32.7

Variable		Frequency	Percent (%)
Campus	Humanities schools	242	30.4
	Medical schools	162	20.4
	Sciences School	391	49.2

Educational level	1^st^ year	111	14.0
	2^nd^ year	195	24.5
	3^rd^ year	91	11.4
	4^th^ year	299	37.6
	5^th^ year	35	4.4
	Higher education MSc & PhD	64	8.1

	Mean ± S.D. (*n* = 727)	Minimum	Maximum

BMI (kg/m^2^)	23.76 ± 4.36	16.24	49.59

Variable		Frequency	Percent (%)
BMI (*n* = 727)	Underweight (18.49 and less)	62	7.8
	Normal weight (18.5–24.9)	508	63.9
	Overweight (25.0–29.9)	157	19.7
	Obese (30.0 and above)	68	8.6

Physical activity	Never	149	18.7
	Less than 30-60 min, one time weekly	174	21.9
	30-60 min, 1-3 times weekly	207	26.0
	30-60 min, 4-6 times weekly	110	13.8
	30-60 min, once daily	85	10.7
	More than 30-60 min, once daily	70	8.8

Smoking status	No	449	56.5
	Yes	346	43.5

GCC: the Gulf Cooperation Council countries; JD: Jordanian dinar; MSc: master's level; PhD: doctor of philosophy level; S.D.: standard deviation; kg: kilograms; cm: centimeters; m: meters; BMI: body mass index; min: minutes.

**Table 2:** Dietary behaviors of the study participants (*N* = 795).

Food items	Frequency of intake (*n* (%))					Quantity of intake (*n* (%))			
	Never (no intake)	1-2 times monthly	1-2 times weekly	3-4 times weekly	1-2 times daily	Never (no intake)	One serving	2-3 servings	More than or equal to four servings
Fruits	23 (2.9)	110 (13.8)	240 (30.2)	207 (26.0)	215 (27.0)	23 (2.9)	474 (59.6)	276 (34.7)	22 (2.8)
Vegetables	19 (2.4)	62 (7.8)	204 (25.7)	254 (31.9)	256 (32.2)	19 (2.4)	329 (41.4)	360 (45.3)	87 (10.9)
Fiber	32 (4.0)	104 (13.1)	277 (34.8)	231 (29.1)	151 (19.0)	32 (4.0)	305 (38.4)	371 (46.7)	87 (10.9)
Onion	153 (19.2)	229 (28.8)	225 (28.3)	148 (18.6)	40 (5.0)	153 (19.2)	521 (65.5)	108 (13.6)	13 (1.6)
Fat & oils	30 (3.8)	83 (10.4)	211 (26.5)	264 (33.2)	207 (26.0)	30 (3.8)	423 (53.2)	284 (35.7)	58 (7.3)
Red meat	75 (9.4)	219 (27.5)	318 (40.0)	159 (20.0)	24 (3.0)	75 (9.4)	468 (58.9)	228 (28.7)	24 (3.0)
Processed meat	245 (30.8)	276 (34.7)	183 (23.0)	78 (9.8)	13 (1.6)	245 (30.8)	335 (42.1)	186 (23.4)	29 (3.6)
Dairy products	32 (4.0)	72 (9.1)	207 (26.0)	313 (39.4)	171 (21.5)	32 (4.0)	416 (52.3)	303 (38.1)	44 (5.5)
French fries	51 (6.4)	222 (27.9)	305 (38.4)	182 (22.9)	35 (4.4)	20 (2.5)	569 (71.6)	175 (22.0)	31 (3.9)

**Table 3:** Levels of knowledge and awareness about CRC risk factors in the study participants (*N* = 795).

Question related to knowledge and awareness about CRC^∗^	Correct answer	Correct (%)
Which group of individuals (males/females) do you think is at greater risk for developing colon cancer?	Males have a greater risk of developing colon cancer.	33.3%
Do you think that diet plays a role in causing CRC?	Yes	63.0%
Do you think that diet plays a role in preventing CRC?	Yes	96.6%
Do you think that CRC is hereditary?	No	51.1%
Risk factors for CRC		% Correct (agree, strongly agree)
Family history of CRC		61.1%
Not being physically active		70.1%
Diabetes		47.9%
Frequent alcohol intake		81.8%
Frequent high-fat intake		79.7%
Increased age		51.9%
Frequent low-fiber intake		72.6%
Being overweight		72.1%
Smoking		76.5%
High frequent intake of red meats		51.3%
High frequent intake of processed meat		77.9%
Low vegetable intake		76.7%
Low fruit intake		73.5%
Frequent high levels of stress		77.9%

Knowledge and awareness score characteristics. Overall score: 12.46 ± 3.93 (out of 18 questions). Percentage score category. <9 (poor knowledge), 16.1%. ≥9 (good knowledge), 83.9%. ^∗^CRC= colorectal cancer.

**Table 4:** Differences in the knowledge and awareness regarding dietary and lifestyle risky or protective behaviors based on the sex variable (*N* = 795).

Behavior	Category	Total, *n* (%)	Female, *n* (%)	Male, *n* (%)	*P* value^∗^
How often do you engage in physical activities?	Less than three days of training/week (infrequent/risky)	530 (66.7%)	410 (51.6)	120 (15.1)	0.0001
	More than/equal to 3 days of activity/week (frequent/protective)	265 (33.3%)	170 (21.4)	95 (11.9)	

What is your usual serving per day of fruits?	Less than two servings/day (infrequent/risky)	683 (85.9)	498 (62.6)	185 (23.3)	0.5240
	More than/equal two servings/day (frequent/protective)	112 (14.1%)	82 (10.3)	30 (3.8)	

What is your usual serving number of vegetables/per day	Less than three servings/day (infrequent/risky)	627 (78.9%)	436 (54.8)	191 (24.0)	0.0001
	More than/equal three servings/day (frequent/protective)	168 (21.1%)	144 (18.1)	24 (3.0)	

What is your usual serving number per day of fiber?	Less than/equal to 1 serving/day (infrequent/risky)	499 (62.8%)	362 (45.5)	137 (17.2)	0.4000
	More than one serving/day (frequent/protective)	296 (37.2%)	218 (27.4)	78 (9.8)	

What is your usual serving number per day of onion?	Less than/equal to 1 serving/day (infrequent/risky)	711 (89.4%)	513 (64.5)	198 (24.9)	0.0850
	More than 1servings/day (frequent/protective)	84 (10.6%)	67 (8.4)	17 (2.1)	

What is your usual serving size per day of dairy products?	Less/equal to 1 serving/day (infrequent/risky)	467 (58.7%)	344 (43.3)	123 (15.5)	0.3250
	More than one serving/day (frequent/protective)	328 (41.3%)	236 (29.7)	92 (11.6)	

What is your usual serving size per day of fat?	More than/equal to 4 servings/day (frequent/risky)	34 (4.3%)	26 (3.3)	8 (1.0)	0.4020
	Less than four servings/day (infrequent/protective)	761 (95.7%)	554 (69.7)	207 (26.0)	

What is your usual serving size per day of red meats?	More than one serving/day (frequent/risky)	98 (12.3)	53 (6.7)	45 (5.7)	0.0001
	Less than/equal to 1 serving/day (infrequent/protective)	697 (87.7)	527 (66.3)	170 (21.4)	

What is your usual serving size per day of processed meats?	More than one serving/day (frequent/risky)	55 (6.9)	33 (4.2)	22 (2.8)	0.0210
	Less than/equal to 1 serving/day (infrequent/protective)	740 (93.1)	547 (68.8)	193 (24.3)	

What is your usual serving size per week of French fries?	More than one serving/week (frequent/risky)	117 (14.7)	81 (10.2)	36 (4.5)	0.1910
	Less than/equal to 1 serving/week (infrequent/protective)	678 (85.3)	499 (62.8)	179 (22.5)	

Smoking	Yes (risky)	357 (44.9)	208 (26.2)	149 (18.7)	0.0001
	No (protective)	438 (55.1)	372 (46.8)	66 (8.3)	

^∗^ *P* value considered significant if it is less than < 0.05.

**Table 5:** The association between the sociodemographic variables and the knowledge and awareness regarding the different risk factors of CRC.

Risk factor	Poor knowledge vs. good knowledge	
	OR (95% CI)	*P* value
Sex (male vs. females)	1.101 (0.662-1.832)	0.4090
Age (18-24 years vs. 25 years and more)	0.931 (0.501-1.730)	0.4630
Marital status (single vs. married)	1.116 (0.511-2.439)	0.4820
Nationality (Jordanian vs. non-Jordanian)	0.647 (0.255-1.640)	0.2460
The school (medical and science vs. humanities)	**0.526 (0.333-0.829)**	**0.0050**
Educational level (undergraduate vs. postgraduate)	1.806 (0.700-4.664)	0.1480
Living place (on-campus vs. out-of-campus)	1.058 (0.355-3.155)	0.5480
Monthly allowance (less than 50 JD vs. more than 50 JD)	1.280 (0.810-2.022)	0.1730
Physical activity (less than three days weekly)	1.070 (0.668-1.712)	0.4390
Fruit daily intake (risky vs. protective)	1.569 (0.782-3.149)	0.1300
Vegetable daily intake (risky vs. protective)	1.081 (0.627-1.864)	0.4500
Fiber daily intake (risky vs. protective)	1.363 (0.850-2.185)	0.1200
Onion daily intake (risky vs. protective)	1.001 (0.505-1.981)	0.5800
Dairy products intake (risky vs. protective)	**1.581 (0.995-2.514)**	**0.0320**
Fat and oil intake (risky vs. protective)	0.748 (0.219-2.550)	0.4520
Red meat intake (risky vs. protective)	1.167 (0.614-2.217)	0.3720
Red, processed meat intake (risky vs. protective)	0.977 (0.398-2.395)	0.5870
French fries intake (risky vs. protective)	1.259 (0.696-2.276)	0.2670
Smoking (smoker)	1.006 (0.628-1.612)	0.5410

Bold values indicate significant associations *P* value < 0.05.

## Data Availability

The data that support the findings of this study are available from the corresponding author upon reasonable request.
